# The efficacy of different alveolar recruitment maneuvers in holmium laser lithotripsy surgery under general anesthesia using a laryngeal mask

**DOI:** 10.1186/s12871-022-01664-y

**Published:** 2022-05-02

**Authors:** Fu-Rong Bai, Hong-mei Li, Ming-liang Yi, Hong Yin, Wei Wu

**Affiliations:** 1 Department of Anesthesiology, Chengdu Fifth People’s Hospital, 33 Mashi Road, Wenjiang District, Chengdu, 611130 China; 2 Department of Anesthesiology, The General Hospital of Western Theater Command, Chengdu, 610083 China

**Keywords:** Laryngeal mask, Lung protective ventilation strategy, Alveolar recruitment maneuvers, Postoperative pulmonary complications

## Abstract

**Background:**

Alveolar recruitment maneuvers (ARMs) is an important part of lung-protective ventilation strategies (LPVSs), but the optimal duration and interval Remain unclear.

**Methods:**

Patients:252 patients who underwent holmium laser lithotripsy surgery and meet inclusion criteria were included and randomized into three groups based on the duration and frequency of ARMs (Regular, one 30 s ARM (RARMs); Improved and intermittent, three 10s ARMs (IARMs); and Control (C), no ARMs).Interventions: Groups R and I received ARMs at 20 cmH2O pressures every 30 min. All patients received the same anesthesia and mechanical ventilation. Measurements:Outcomes included heart rate and mean arterial pressure changes during ARMs and postoperative pulmonary complications (PPCs) within the first 7 postoperative days.

**Main results:**

Incidences of PPCs in groups R(7.1%) and I (5.0%)were slightly lower than those in group C (8.9%).This indicated the potential to reduce lung injury. Heart rate and mean arterial pressure fluctuations during ARMs were significantly higher in groups R and I than in group C (*P* < 0.01). The rate of blood pressure decrease was significantly higher in group R than in group I (*P* < 0.01).

**Conclusions:**

IARMs can reduce cycle fluctuations than RARMs in patients Undergoing holmium laser lithotripsy surgery with laryngeal mask general anesthesia. Low tidal volume ventilation and low PEEP combined with ARM did not significantly reduce the incidence of PPCs in healthy lung patients, but tended to reduce lung injury.

**Trial registration:**

The study was registered on the Chinese Clinical Trial Registry.

(ChiCTR2000030815,15/03/2020). This study was approved by the ethics committee of Chengdu Fifth People’s Hospital with approval number(2020–005(Study)-1).

**Supplementary Information:**

The online version contains supplementary material available at 10.1186/s12871-022-01664-y.

## Background

With the development of comfort medicine and enhanced recovery after surgery (ERAS), the application of laryngeal mask general anesthesia in ureteral calculi holmium laser lithotripsy has been widely used in China. Usually, the calculi move with respiration, leading to interference with surgery; to reduce the interference, the anesthesiologist often needs to reduce the tidal volume or briefly stop breathing during the operation. Although such respiratory management can effectively reduce the impact of breathing movement during surgery, it may also increase postoperative pulmonary complications (PPCs) such as hypoxemia, atelectasis, and hypercapnia [1, 2]. PPCs cause considerable harm to patients and are the main cause of related complications [3]. Studies have shown that lung protective ventilation strategies (LPVS) can improve postoperative lung function and reduce the incidence of PPCs in patients undergoing surgery,it include low tidal volume ventilation, positive end-expiratory pressure (PEEP), pulmonary re-extension, low inhaled oxygen concentrations, and permissible hypercapnia [4–6]. Low tidal volume ventilation can lead to atelectasis [2]; however, collapsed alveoli can be reversed by ARM [7], and appropriate PEEP can maintain open alveoli [8]. There is currently no standard for the duration of ARM, with prior procedures using durations of 10 to 50 s at different pressures [9–13]. Similarly, the use of laryngeal mask anesthesia for lung protection has rarely been studied. We hypothesized that patients undergoing laryngeal mask general anesthesia employed IARMs was safer and would reduce the incidence of PPCs.

## Methods

### Participants

The study was registered on the Chinese Clinical Trial Registry (ChiCTR2000030815,15/03/20). This study was approved by the ethics committee of Chengdu Fifth People’s Hospital with approval number(2020–005(Study)-1). The subject participant provided written consent. All methods were carried out in accordance with the relevant guidelines and regulations. Risk and benefits were discussed with patients and relatives, and informed consent forms were obtained from all participants prior to enrollment. 252 participants were selected from 653 participants for scheduled transurethral ureteroscopic holmium laser lithotripsy with general Anesthesia from April 2020 to December 2020. Inclusion criteria were age 18 to 65 years with ASA Class I–III, a BMI of 18–30 kg/m^2^, an expected operation time of 1–3 h, and no contraindications to laryngeal mask placement. Exclusion Criteria were a history of surgery or invasive mechanical ventilation within 2 weeks, respiratory failure or sepsis, heart failure, liver and kidney insufficiency,pregnancy, alcohol abuse, morphine addiction, and mental system disorders.

Withdraw Criteria were Laryngeal mask factor (experienced anesthesiologist failed to place 3 times,laryngeal mask leakage caused by various intraoperative factors), Surgery has changed,the experimental operation was not carried out for various reasons. Patients were randomized into three groups based on random numbers: One 30s ARM interval (Regular group, R, *n* = 84), three 10 s ARM intervals (Improved group, I, *n* = 84), and no ARM (Control group, C, *n* = 84). The random numbers were generated by a computer and randomly divided into three groups in a ratio of 1:1:1 into opaque, sealed envelopes, which were then passed by a non-participant investigator to the anesthesiologist, who administered the anesthetic for patients.

People including investigators, patients, staff in the ward and postoperative care units (PACU)) were unaware of the grouping. Postoperatively,all data were obtained by investigators who did not know the groups.

### Anesthesia protocol

All patients fasted for 8 to 12 h prior to general anesthesia, and no preoperative medication was administered. Upper extremity intravenous (IV) access was established, and 5–10 ml/kg crystalloids were administered before anesthesia. Echocardiography (ECG), heart rate (HR), non-invasive blood pressure (NIBP), oxygen saturation (SpO2), and EtCO2 were monitored. A radial artery puncture catheter was placed under local anesthesia to monitor intraoperative arterial pressure and harvest samples for blood gas analysis. Anesthesia was induced by administering midazolam (0.03 mg/kg), sufentanil (0.3 ug/kg), propofol (1–1.5 mg/kg), and cisatracurium (0.15 mg/kg).

Laryngeal masks were selected based on weight: size 3 for <50 kg, size 4 for 50–70 kg, and size 5 for >70 kg. Mechanical ventilation was established after the oral insertion of the laryngeal mask. Sevoflurane was inhaled for sedation, and remifentanil maintenance infusion rate of 0.05 ~ 0.1 μg kg − 1 min − 1 for anesthesia maintenance. The Bispect ral index (BIS) value was maintained between 40–60 s, the average arterial pressure (MAP) was maintained between 70–100 mmHg, and HR was maintained between 50–100 beats per minute (bpm).Ringer’s fluid was instilled intravenously (5–10 ml/kg/h) to maintain body fluids. A blood transfusion was considered for blood loss >30% of the total blood volume or a hemoglobin level < 7 g/dl; otherwise, colloidal fluid was used to replace the volume of blood lost. All anesthetics were ceased after the double-J tube was placed. The laryngeal mask was removed when spontaneous breathing and swallowing reflexes were reestablished, normal tidal volume was recovered, and patients responded to verbal commands with their eyes open. The patient was transported back to the recovery ward after complete awakening, and all vital signs were normal.

### Mechanical ventilation protocol

A tidal volume of 6 ml/kg, respiratory frequency of 12–18 breaths per minute, inspiration to expiration ratio of 1:2, and PEEP of 5 cmH2O were used in all groups. All patients were administered 50% oxygen at 2 L/min total flow, and respiratory rates were adjusted to maintain PaCO2 at 35–45 mmHg. Groups R and I received ARM every 30 min during surgery, whereas Group C did not receive ARM. ARMs were conducted by adjusting the pressure valve to 20 cmH2O, followed by adjusting the manually controlled breathing valve, and squeezing the air bag to maintain airway pressure. If the cyclic fluctuation during the period of the ARM exceeds 20% before ARM, the test will be terminated.

### Outcomes

Primary outcomes included the incidence of PPCs within days, and changes in heart rate and mean arterial pressure during ARM. Secondary outcomes included the duration of postoperative hospital stay, patient satisfaction, and postoperative mortality within 30 days. We also recorded the mechanical ventilation duration and extubation time (from the end of the operation to the time of laryngeal mask removal), and analyzed arterial blood gases and airway peak pressure at T1 (preoperative), T2 (1 h after ventilation), and T3 (postoperative). We monitored postoperative symptoms including cough, expectoration, lung auscultation, fever, and complications associated with laryngeal mask placement.

### Sample size and statistical methods

We used the excellent effect test for multiple proportion (provided by the Power Analysis and Sample Size,PASS) to calculate the sample size. According to the literature data, the incidence of PPCs was between 5 and 50% in different types of surgery and 30% relative reduction in PPCs would have clinical significance. However, the incidence of this type of surgery is rarely reported, so, we assume that the incidence of PPCs in the control group was 30 and 15% in the experimental group R and 10% in the experimental group I,and proportion among the groups was 1:1:1. The estimated sample size was 68 per group,which provide 80% power, with two-sided level ofα = 0.05. Taking into account the dropout rate of 20%, so we planned to enroll 252 patients (84 for each group) in all.

We analysed outcome data with SPSS version 26.0software.Statistical significance was set at *P* < 0.05.Statistical description was provided for baseline data,such as age, sex, BMI, ASA, smoking status, Chest X - ray examination. The quantitative data with a normal distribution were expressed as the mean ± standard deviation, data satisfying the homogeneity of variance were compared using one-way analysis of variance (ANOVA). Fisher’s least significant difference-t test (LSD-t test) was used for post hoc analysis. whereas non-parametric test were used for non- normally distributed. The categorical data were presented as the number (percentage) and used the chi-square test.

The estimated sample size was 84 cases in each group, and the sample size was finally included in the analysis (56, 60, 67). The reasons for some patients not included in the analysis were as follows: Duration of surgery<1 h;Laryngeal mask placement failed and was replaced with endotracheal intubation;Surgery has changed;the experimental operation was not carried out for various reasons;the experimental operation was not carried out for various reasons. If these patients were included in the analysis, it would affect the accuracy of our main outcome. Therefore, these patients were not included and we compared the dropout rates among the three groups, Chi-square test was performed and there was no significant difference (*P* = 0.156).

## Results

### Patients characteristics

**Fig. 1:**
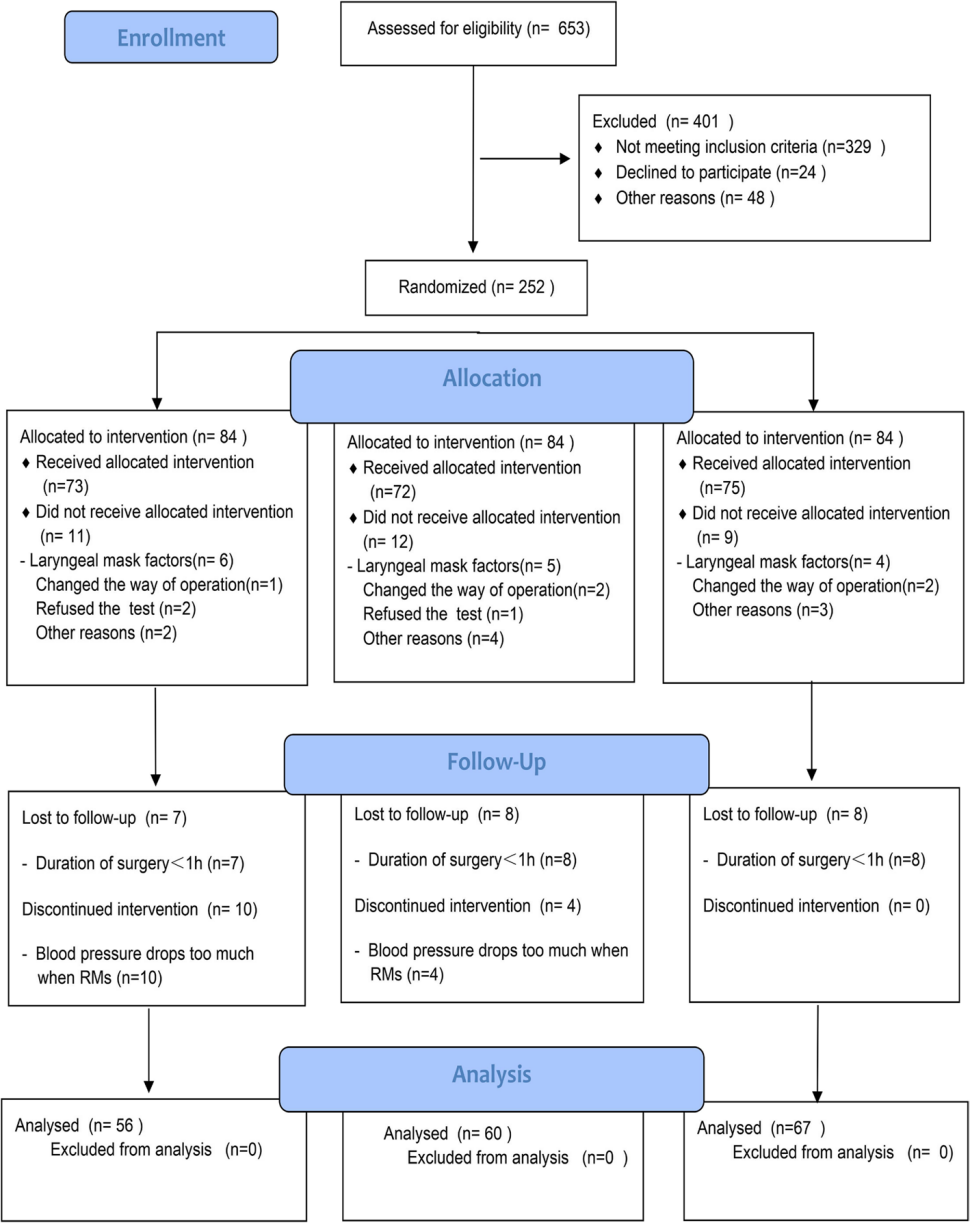
Flow diagram

Two hundred fifty two patients were randomized,following assessment with exclusion criteria, 183 individuals were included in the analysis (Fig. 1). There were no statistically significant differences among the three groups in terms of sex, age, body mass index (BMI), ASA class, history of smoking, and preoperative chest X-ray characteristics (Table 1).

**Table 1:** Preoperative baseline characteristics of 252 patients

Indicator	R Group (*n* = 84)	I Group (*n* = 84)	C Group (*n* = 84)	*F/χ2*value	*p* value
Age (year; mean ± SD)	42.40 ± 13.37	43.87 ± 13.85	41.08 ± 13.24	0.892	0.409a
Male [n(%)]	66 (78.5)	64 (76.2)	65 (77.3)	0.136	0.934b
BMI (kg/m2; mean ± SD)	22.20 ± 2.88	22.51 ± 2.88	22.73 ± 2.74	0.764	0.485a
ASA-II [n(%)]	36 (42.9)	41 (48.8)	42 (50.0)	0.987	0.61b
Smoking [n(%)]	35 (41.6)	43 (51.1)	37 (44.1)	1.663	0.435b
Abnormal Chest X-ray [n(%)]	43 (51.2)	50 (59.5)	48 (57.1)	1.256	0.486b

*Legend*: *BMI* Body Mass Index, *ASA-II* American Society of Anesthesiologists physical status classification-II, ^a^ One-way ANOVA test, ^b^ Chi-square test

### Changes of the circulatory system

**Fig. 2:**
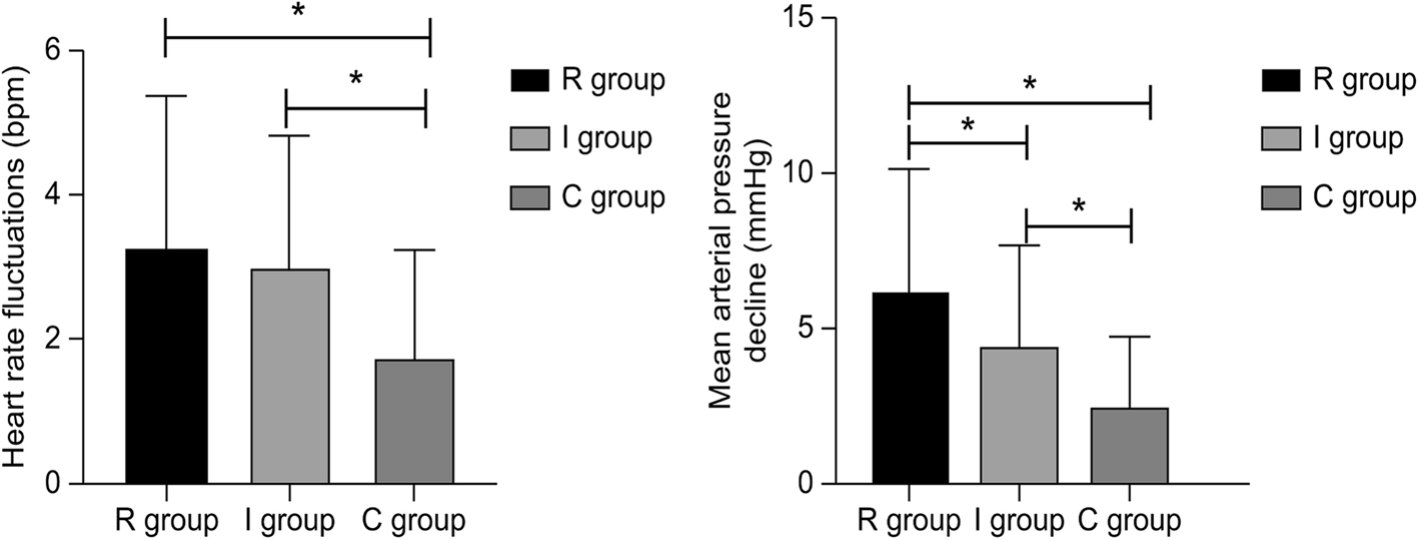
Cardiovascular system changes during ARM in the 3 groups Legend:“*“Comparison between the two groups, *P* < 0.05

The decrease in mean arterial pressure was significantly higher in group R than in group I (6.18 ± 4.10 mmHg vs. 4.47 ± 3.35 mmHg, *p* < 0.01). Heart rate fluctuations during ARM has no significant difference between groupR and groupI (3.21 ± 2.15 bpm vs 3.07 ± 1.81 bpm, *P* = 0.114). Fluctuations in heart rate and blood pressure were not obvious in group C because ARMs were not performed, and the changes in heart rate and mean arterial pressure were significantly higher in groups R and I than in group C *(P* < 0.01). These results are presented in Table 2 and Fig. 2.

**Table 2:** Cardiovascular system changes during ARM in the 3 groups

Indicator	Rgoup (*n* = 56)	Igoup (*n* = 60)	Cgoup (*n* = 67)	*F*	*P*
Mean arterial pressure Decline in value (mmHg)	6.18 ± 4.10	4.47 ± 3.35a	1.93 ± 1.71ab	28.618	*P*<0.001
Heart rate fluctuation value (bpm)	3.21 ± 2.15	3.07 ± 1.81c	1.51 ± 1.11ab	19.283	*P*<0.001

One-way ANOVA test and LSD test was used

*Legend*: ^a^Compared with group R, *P* < 0.05;

^b^compared with group I, *P* < 0.05,

^c^Compared with group R, *P* = 0.644

### Postoperative follow-up and PPCs within the first 7 days after surgery

Thirteen patients developed PPCs (7.1%); four occurred in group R (7.1%), three occurred in group I (5.0%), and six occurred in group C (8.9%). The incidence of PPCs was slightly higher in group C than in groups R and I, but there was no significant difference in PPCs between groups (*P* > 0.05). There were no statistically significant differences between the groups with respect to complications of laryngeal mask placement and days in hospital. No deaths occurred during hospitalization (Table 3).

**Table 3:** Outcomes within the first 7 days after surgery

Indicator	Rgroup (*n* = 56)	Igroup (*n* = 60)	Cgroup (*n* = 67)	*F/χ2*value	*p* value
PPCs [n(%)]	4 (7.14)	3 (5.00)	6 (8.95)	0.751	0.687a
Pneumonia [n(%)]	2 (3.57)	1 (1.66)	1 (1.49)	0.729	0.694a
atelectasis [n(%)]	1 (1.78)	1 (1.66)	2 (2.98)	0.318	0.853a
hyoxemia [n(%)]	1 (1.78)	1 (1.66)	3 (4.47)	1.213	0.545a
Laryngeal mask related complications [n(%)]					
Voice hoarse	2 (3.5)	2 (3.3)	2 (2.9)	0.034	0.983a
Sore throat	6 (10.7)	7 (11.6)	6 (8.9)	0.260	0.878a
Mucosal hemorrhage	5 (8.9)	4 (6.6)	4 (5.9)	0.430	0.806a
Cough	3 (5.3)	4 (6.6)	3 (4.5)	0.295	0.863a
fever	5 (8.9)	6 (10.0)	6 (8.9)	0.053	0.974a
Postoperative complications [n(%)]					
Abnormal auscultation of the lungs	1 (1.7)	1 (1.6)	2 (2.9)	0.318	0.853a
Patient satisfaction [n(%)]					
Satisfied	45 (80.3)	47 (78.3)	56 (83.5)	0.579	0.749a
Postoperative hospital stay (day)	3.52 ± 1.21	3.48 ± 1.42	3.56 ± 1.37	0.052	0.974b

There was no significant difference among the three groups in the above indicators

^a^Chi-square test,

^b^One-way ANOVA test

### Other outcomes

There were no statistically significant differences among the groups with respect to intraoperative fluid administration or loss of body fluids, mechanical ventilation duration, or extubation time (Table S1).

There were no statistically significant differences among the three groups in terms of airway peak pressure or arterial blood gas analyses of PH, Oxygenation index (OI), and PaCO2 at T1, T2, and T3 (Table S2).

## Discussion

Although there was no statistically significant improvement in PPCs after ARM, the incidence was lower in the improved group, indicating the potential to reduce lung injury. This result requires further confirmation in clinical studies with larger sample sizes. Nonetheless, the incidence of PPCs was lower than previously reported in the Literatures [14–17]. This could be attributed to the small sample size, differences in inclusion criteria, with the prior LPVS trials including patients with acute respiratory distress syndrome (ARDS) [18, 19], elderly patients [20], and patients undergoing thoracic surgery [21], versus the healthy lung patients in this study. Additionally, it is possible that the duration of mechanical ventilation was insufficient. In order to reduce retrograde infection, urethral injury, and water poisoning, the operation time for this type of surgery is typically less than 2 h in our hospital, meaning the mechanical ventilation duration is generally not more than 3 h. Fourth, diagnostic criteria may differ.

In this study, intermittent ARM (IARM) had less influence on circulation than continuous ARM. In clinical practice, intermittent ARM may be safer, but certain patients need to more time to open alveoli, including obese patients and patients undergoing thoracoscopic or laparoscopic procedures. Studies have shownthat the laryngeal mask has a protective effect on the lungs [22, 23]. The laryngeal mask is a supraglottic ventilation device that widely used in short operations and emergency airway rescue because it is simple to operate, has small injury risks, Does not require special positioning,and has good patient tolerance [23, 24]. The third-generation double-tube laryngeal mask used in this study includes a gastric tube, which greatly improves its effectiveness and safety. The laryngeal mask was successfully placed after anesthesia in 94% of patients, and there were no statistically significant differences in the number of complications associated with laryngeal mask placement and removal among the three groups.

The application of LPVS in this study included selection of an optimal tidal volume. The tidal volume should be chosen based on comprehensive consideration of patients’ lung function, lung compliance, thoracic compliance, and functional residual gas, and the tidal volume is calculated based on the patient’s predicted body weight (PBW) [25–27]. Excessive tidal volume can lead to excessive expansion of the alveoli and cause "volume injury" [26]. Excessive tidal volume also increases airway pressure, the possibility of gas entering the stomach, and air leakage. However,a low tidal volume can lead to insufficient ventilation resulting in atelectasis, ventilation/blood flow disorders, and respiratory acidosis [2]. Consensus among experts recommends a tidal volume of 6–8 mL/kg PBW, which was consistent with the tidal volume used in this study. Additionally, PEEP can maintain the alveoli in the open state during ventilation, and improve oxygen and pulmonary compliance.

Prior research indicates that PEEP ≥5 cmH2O can improve lung compliance, enhance patient oxygenation, and reduce the occurrence of PPCs [8]. Compared with PEEP ≤2 cmH_2_O, PEEP >12 cmH_2_O increased the risk of increased peak airway pressure and hemodynamic fluctuations [28]. Experts recommend at least 5 cmH2O, which was used in our study. Alveolar recruitment maneuvers performed at 30-min intraoperative intervals have been shown to improve trans-pulmonary pressure, reopen collapsed alveoli caused by insufficient ventilation, improve oxygen and lung compliance, and reduce the mortality of ARDS patients [29] .If the systolic blood pressure is less than 80 mmHg, or arrhythmia occurs, the ARM should be terminated [18].

There are several limitations associated with this study. It was a single-center study, with a small sample size, and only included healthy lung patients. Additionally, there was no comparison to other ventilation modalities, and strategies for perioperative lung protection should be multifactorial. The effectiveness of LPVS on PPCs was only assessed using one set of variables. A multicenter study with a substantially larger sample size is required to confirm our findings.

## Conclusions

In conclusion, Low tidal volume ventilation and low PEEP combined with ARM did not significantly reduce the incidence of PPCs in healthy lung patients, but tended to reduce lung injury. IARMs can reduce cycle fluctuations than RARMs in patients Undergoing holmium laser lithotripsy surgery with laryngeal mask general anesthesia.

## Supplementary Information

**Additional file 1: Table S1.** Mechanical ventilation duration or extubation time.

**Additional file 2: Table S2.** Intraoperative ventilation indexes of patients in three groups.

## Data Availability

The datasets used and analyzed in the current study are available from the corresponding author upon reasonable request.

## Abbreviations

ASA: American Society of Anesthesiologists
VILI: Ventilation related lung injury
LPVS: Lung protective ventilation strategy
PPCs: Postoperative pulmonary complications
PEEP: Positive end-expiratory pressure
ARMs: Alveolar recruitment maneuvers
IARM: Improved alveolar recruitment maneuvers
RARM: Regular alveolar recruitment maneuvers
ICU: Intensive Care Unit
BMI: Body mass index
PBW: Predicted body weight
OI: Oxygenation index
BIS: Bispect ral index
ECG: Echocardiography
HR: Heart rate
NIBP: Non-invasive blood pressure
SpO2: Oxygen saturation
