# Supplementary material for: The efficacy of different alveolar recruitment maneuvers in holmium laser lithotripsy surgery under general anesthesia using a laryngeal mask
Source: BMC Anesthesiol. 2022 May 2;22:134. doi: 10.1186/s12871-022-01664-y (PMC9063066; doi:10.1186/s12871-022-01664-y)
Supplement: Supplementary file 1 — Additional file 1: Table S1. Mechanical ventilation duration or extubation time. [file 12871_2022_1664_MOESM1_ESM.pdf]

Table S1:Mechanical ventilation duration, or extubation time.

Indicator	R Group（n=56）	I Group (n=60)	C Group (n=67)	*F* value	*p* value
Blood loss（mL）	7.66±1.70	7.55±1.65	7.49±1.70	0.154	0.857
Total fluids （mL）	1111.07±254.79	1044.70±213.52	1119.94±235.27	1.878	0.156
Mechanical ventilation time (min)	126.96±23.23	121.82±20.51	127.15±25.20	1.038	0.356
Extubation time(min)	9.91±2.21	9.25±2.29	9.01±2.23	2.538	0.082

One-way ANOVA was used for all indicators .
