# Supplementary material for: The efficacy of different alveolar recruitment maneuvers in holmium laser lithotripsy surgery under general anesthesia using a laryngeal mask
Source: BMC Anesthesiol. 2022 May 2;22:134. doi: 10.1186/s12871-022-01664-y (PMC9063066; doi:10.1186/s12871-022-01664-y)
Supplement: Supplementary file 2 — Additional file 2: Table S2. Intraoperative ventilation indexes of patients in three groups. [file 12871_2022_1664_MOESM2_ESM.docx]

Table S 2. Intraoperative ventilation indexes of patients in three groups

| group | Point in time | Ppeak(cmH_2_O) | PH | OI | PaCO_2_ |
| --- | --- | --- | --- | --- | --- |
| Rgroup（n=56） | T1 |  | 7.41±0.036 | 453.13±26.84 | 39.39±3.25 |
|  | T2 | 15.66±1.861 | 7.36±0.028 | 460.11±36.18 | 39.32±3.76 |
|  | T3 | 16.25±1.632 | 7.38±0.036 | 448.63±37.91 | 41.09±3.55 |
| Bgroup（n=60） | T1 |  | 7.40±0.046 | 449.25±33.101 | 39.85±3.49 |
|  | T2 | 15.90±1.811 | 7.36±0.028 | 454.48±33.354 | 39.37±3.84 |
|  | T3 | 16.27±1.982 | 7.37±0.040 | 456.45±30.187 | 41.92±3.44 |
| Cgroup（n=67） | T1 |  | 7.40±0.048 | 451.33±30.66 | 39.96±2.92 |
|  | T2 | 15.93±2.017 | 7.36±0.027 | 448.72±35.03 | 39.67±3.54 |
|  | T3 | 16.33±1.862 | 7.38±0.041 | 446.88±28.09 | 41.88±3.39 |

T1:preoperative, T2 :1 h after ventilation, T3 :postoperative， Ppeak : Peak airway pressures, PH:Potential of hydrogen, OI: Oxygenation index,PaCO2: partial pressure of carbon dioxide in arterial blood.One-way ANOVA test and LSD test was used( *P*＞0.05) .
